# A Case Series of Statin-Induced Necrotizing Autoimmune Myopathy

**DOI:** 10.7759/cureus.21613

**Published:** 2022-01-25

**Authors:** Alisha Sharma, Clio Musurakis, Nur un nisa Nabil, Bidhya Poudel, Angkawipa Trongtorsak

**Affiliations:** 1 Internal Medicine, AMITA Health Saint Francis Hospital, Evanston, USA; 2 Endocrinology , Diabetes and Metabolism, Chicago Medical School - Rosalind Franklin University, North Chicago, USA; 3 Primary Care, Community Hospital Anderson, Anderson, USA

**Keywords:** idiopathic rhabdomyolysis, statin-induced myopathy, proximal weakness, statin, sinam, statin-induced necrotizing autoimmune myopathy

## Abstract

The use of statins has been increasing over the past decade for the primary and secondary prevention of cardiovascular disease worldwide. Subsequently, various side effects have also been unfolding. Muscle-related side effects secondary to statins range from myalgia to rhabdomyolysis and need close monitoring for early detection. Statin-induced necrotizing autoimmune myopathy (SINAM) in particular is unique given its pathophysiology, trigger factor, genetic predisposition, and aggressive management strategy. We present two cases of SINAM and discuss the clinical aspects of diagnosis, investigation, and management. Statin-induced necrotizing autoimmune myopathy usually presents with proximal myopathy along with increased creatinine kinase (CK) levels which do not resolve with only statin discontinuation. Diagnosis should be made with biopsy and 3-hydroxy-3-methylglutaryl-coenzyme A reductase (HMGCR) antibody detection. The investigation should also be directed to rule out other etiology of proximal myopathy. In most cases, rechallenge with a statin is unsuccessful and immunosuppressive treatment is essential.

## Introduction

Statin-induced necrotizing autoimmune myopathy (SINAM) is a rare side effect with the incidence of two cases per million per year [1]. It is much more severe compared to self-limited statin-induced myopathy and is associated with significant symmetric proximal muscle weakness, markedly elevated creatine kinase (CK) level, and persistent symptoms despite statin discontinuation. The sensitivity and specificity of the 3-hydroxy-3-methylglutaryl-coenzyme A reductase (HMGCR) antibody are 94.4% and 99.3%, respectively. It should be utilized along with the biopsy when suspicion of the condition exists [2]. Though uncommon, its early detection and proper management are crucial to halt the disease progression and achieve remission. Here, we present two cases that highlight the key points to recognize this uncommon condition early.

## Case presentation

Case 1

A 67-year-old male with a significant past medical history of hypertension, dyslipidemia, meningioma, and squamous cell carcinoma of the lung presented with a one-month history of proximal muscle weakness. He was not able to get up from a sitting position without support and had difficulty raising his hand above his head. He had no sensory changes, paresthesia, headache, visual changes, flu-like symptoms, and had not travelled anywhere recently. He had been in remission and was being monitored for squamous cell carcinoma of the lung. The physical examination revealed motor power of 3/5 on bilateral upper extremity on shoulder abduction and 4+/5 on bilateral hip flexors which were consistent with proximal muscle weakness. Deep tendon reflexes were 2+ and symmetric. There were no sensory deficits. There was no tenderness elicited on any muscle group. Cranial nerves were intact. He had no rashes. The rest of the physical exam was unremarkable. 

**Figure 1:**
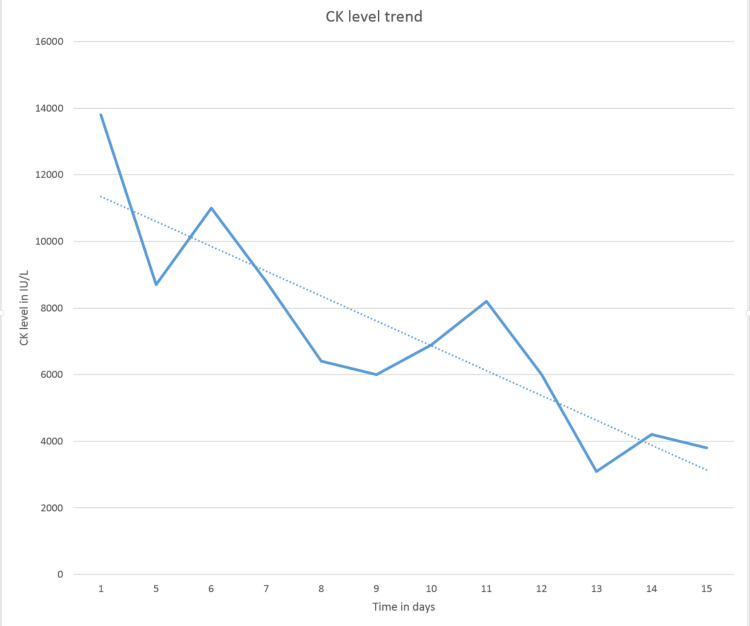
CK level trend since admission to discharge CK: Creatine kinase

Lab results revealed a high CK level (13,635 IU/L: reference range of 30-223 IU/L) and a high aldolase level (93 U/L: reference range of 1.2-7.6 U/L). Although the patient had been noncompliant with medications in the past, he had been taking atorvastatin 40 mg regularly for the last month. Statin was discontinued and work up for paraneoplastic syndrome was done. He continued to have muscle weakness with minimal improvement in CK levels (Figure 1 ). The persistence of weakness despite discontinuation of statin had cast doubt on statin-induced myopathy and an extended myositis panel with specific antibodies (Table 1) were ordered. 

**Table 1:** Myositis antibody panel MDA-5: Melanoma differentiation-associated gene 5, SRP: Signal recognition particle, NXP-2: Nuclear matrix protein-2

Antibody	Result
Fibrillarin (U3 RNP) Ab	Negative
Liver-kidney microsome-1 Ab	Negative
MDA-5 antibody	Negative
SSA 52 Ro ENA Ab IgG	Negative
SSA 60 Ro ENA Ab IgG	Negative
Ribo Prot U1 ENA Ab	Negative
Jo-1 (histidyl -tRNA synthetase) Ab	Negative
PM/SCL 100 Ab	Negative
Mi-2 (nuclear helicase protein) Ab	Negative
PL-7 (threonyl-tRNA synthetase) Ab	Negative
PL-12 (alanyl-tRNA synthetase) Ab	Negative
P155/140 (FIF1-gamma) Ab	Negative
Ku Antibody	Negative
U2 sn (small nuclear) RNP Ab	Negative
EJ (glycyl-tRNA synthetase) Ab	Negative
SRP (signal recognition particle) Ab	Negative
OJ (isoleucyl-tRNA syn.) Ab	Negative
SAE1 (SUMO activating enzyme) Ab	Negative
NXP-2 (nuclear matrix protein-2) Ab	Negative
MDA5 (CADM-140) Ab	Negative
NXP-2 (nuclear matrix protein-2) Ab	Negative
TIF -1 gamma (155 kDA) Ab	Negative

**Figure 2:**
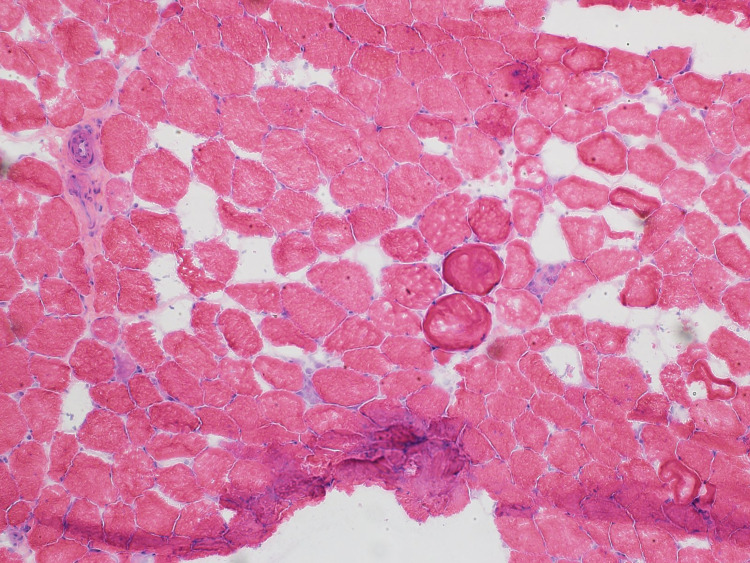
H&E staining showing variation in muscle fibre size with scattered necrotic and regenerating muscle fibres H&E staining: Hematoxylin and eosin stain

**Figure 3:**
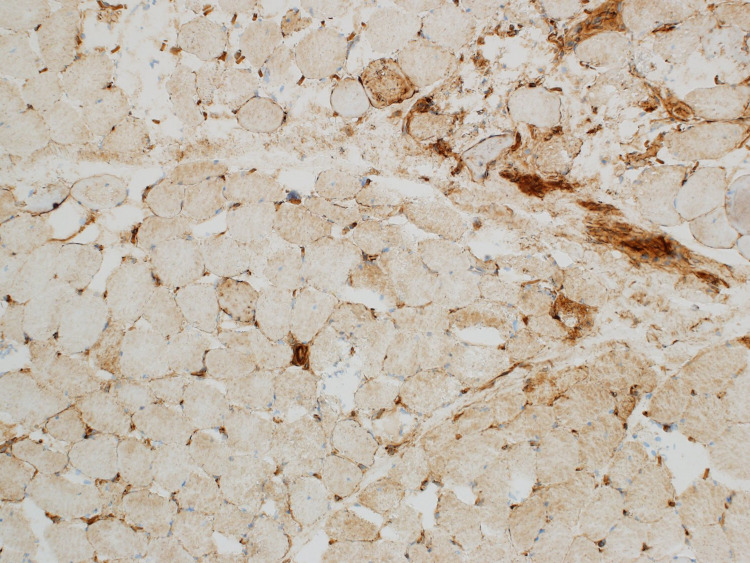
Patchy sarcolemmal/sarcoplasmic MHC class 1 expression MHC: Major histocompatibility

**Figure 4:**
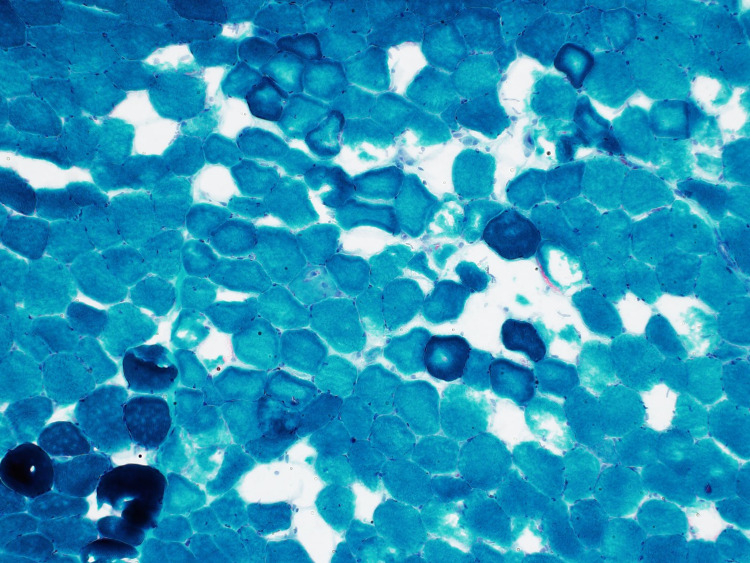
Trichrome staining showing necrotic muscle fibres which are seen with a darker staining pattern

The patient was started on prednisone 60 mg and a muscle biopsy was scheduled. He improved on steroids and was discharged on prednisone 40 mg with outpatient follow-up at the neurology clinic. Electromyography (EMG) done as an outpatient showed significant primary muscle disease with proximal muscle response of reduced amplitude, normal distal latency, and normal conduction velocity. Muscle biopsy from the left quadricep muscle revealed scattered regenerating and necrotic muscle fibres without chronic inflammation. Given the presence of a diffuse increase in muscle fibre staining for major histocompatibility complex class 1 (MHC1), immune-mediated necrotizing myopathy was suspected (Figure 2-4).

The HMGCR antibody test was ordered which turned out to be positive (110 U: reference range is 0-19 U) while the results for paraneoplastic (Table 2) and myositis panel (above in Table 1) came back negative. The diagnosis of SINAM was made and prednisone 40 mg was continued for a total of 10 days. It was then tapered to 20 mg over two weeks. The patient on a two-week follow-up had some improvement in muscle strength of the upper limb with the power of 4/5 on shoulder abduction but there was a persistent weakness of the hip flexors. He was then referred to rheumatology for evaluation on starting immunosuppressant medication. He was initially started on methotrexate 2.5 mg weekly which was then increased to 10 mg after one month due to minimal response. He did well on methotrexate 10 mg weekly and had improvement in proximal muscle strength regaining his baseline power at the four-month follow-up. He continued methotrexate 10 mg weekly and prednisone 5 mg daily for a total of six months. He also had a repeat HMGCR antibody done at six months which was negative with a value of 18 U. Prednisone was then tapered over one month and methotrexate was discontinued.

**Table 2:** Paraneoplastic reflexive panel

Antibody	Result
CV2.1 Antibody	<1:10 (Negative)
Purkinje Cell/Neuronal Nuclear IgG	Not detected
Neuronal Antibody (Amphiphysin)	Not detected

Case 2

A 57-year-old male with a significant past medical history of hypertension, hyperlipidemia, and type 2 diabetes mellitus presented to the hospital with generalized weakness. His weakness started three weeks before he arrived at the hospital. He had associated muscle stiffness which improved throughout the day and with physical activity. He had difficulty climbing stairs and lifting objects over his head. He had no muscle pain, paresthesia, vision changes, difficulty with swallowing, or speech difficulties. He had no recent upper respiratory tract infection, gastrointestinal symptoms, recent travel, or a recent visit to the outdoors. The patient did not start any new medications recently but had been taking atorvastatin 40 mg for the past five years. Other medications were insulin, lisinopril, and metformin. When he arrived at the hospital his vital signs were stable. His physical exam revealed decreased proximal strength of the bilateral upper and lower extremities; right hip flexor 4+, left hip flexor 4-, shoulder abduction 4+ bilaterally. The tone was normal and symmetric. There was no pronator drift or tremor. Deep tendon reflexes (DTRs) were 2+ and symmetric. He was able to perform heel and toe walking but was unable to perform single-leg hop due to weakness. There were no sensory deficits. Cranial nerves were intact. He had no rashes. The rest of the physical exam was unremarkable.

**Figure 5:**
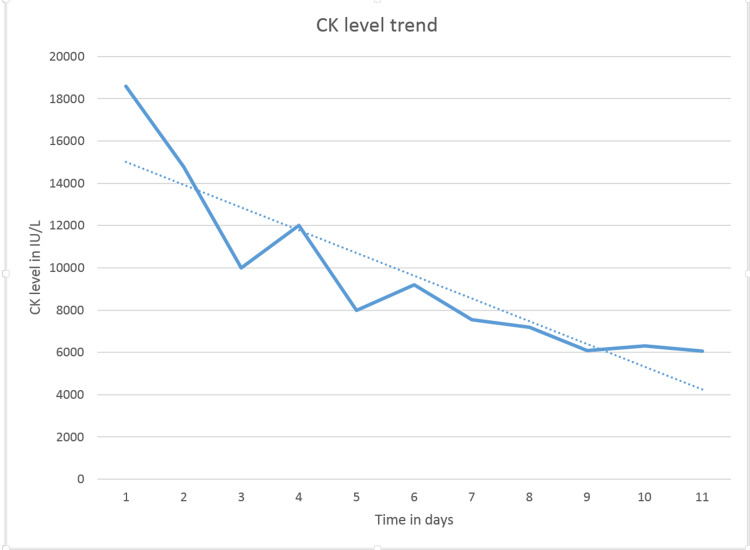
CK level trend since admission to discharge CK: Creatinine kinase

**Figure 6:**
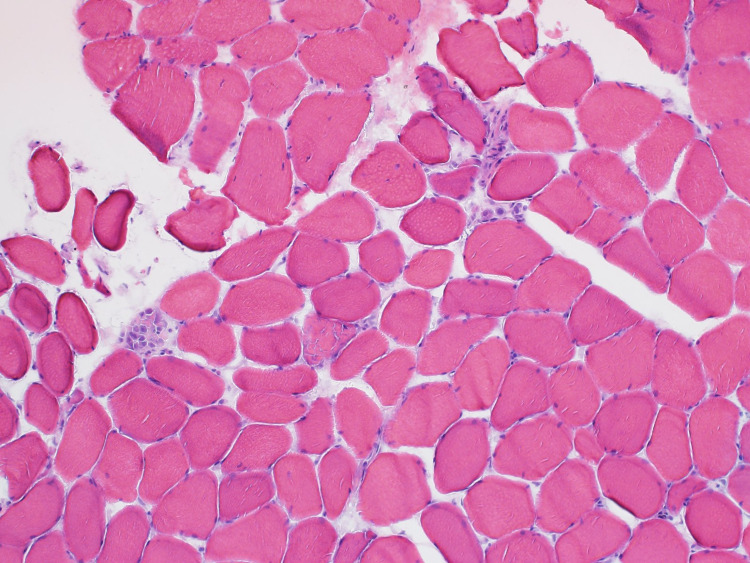
H&E stain showing a few necrotic muscle fibres H&E staining: Hematoxylin and eosin stain

**Figure 7:**
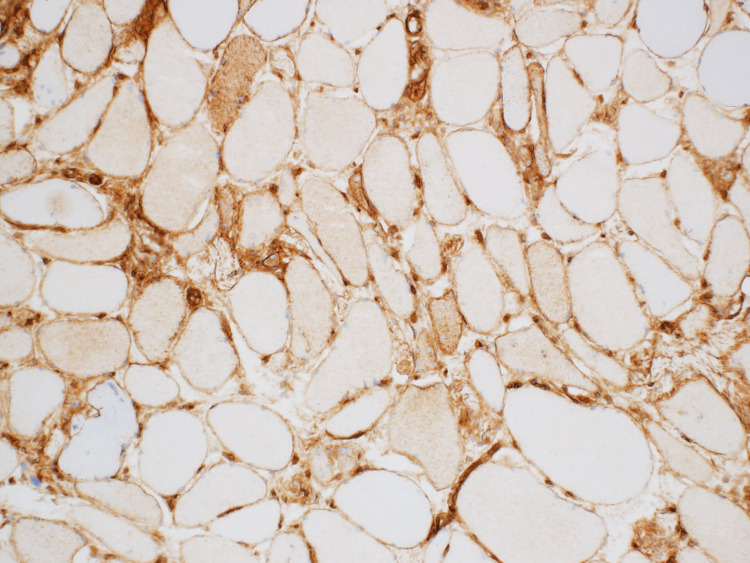
Sarcolemmal/sarcoplasmic MHC class I expression MHC: Major histocompatibility

Lab workup included CK levels which were high (18351 IU/L) as well as a high Aldolase ( 157 IU/L). Comprehensive metabolic panel (CMP) was remarkable for aspartate aminotransferase (AST) 284 IU/L, and alanine transaminase (ALT) 421 IU/L. The complete blood count (CBC) with differential was unremarkable. Lactic acid dehydrogenase (LDH) was also elevated with the value of 1415 IU/L. Urinalysis showed amber coloured urine, large blood, and mild proteinuria. Erythrocyte sedimentation rate (ESR), c-reactive protein (CRP), and thyroid-stimulating hormone (TSH) were within normal limits. The test for COVID PCR was negative. Antinuclear antibodies (ANA), anti-neutrophilic cytoplasmic autoantibody (ANCA) vasculitis profile, rheumatoid factor, anti-Jo-1, anti-scleroderma antibody, SSA and SSB antibody, hepatitis acute panel, extended myositis, and paraneoplastic reflexive panel were negative. His CK levels remained above 6000 for days despite aggressive hydration and discontinuation of statin (Figure 5). He had a muscle biopsy which revealed necrotizing myopathy (Figure 6 and Figure 7). The 3-hydroxy-3-methylglutaryl coenzyme A (HMG-CoA) reductase antibody returned positive (>200 units) confirming the diagnosis of SINAM He was started on prednisone 40 mg which was tapered to 10 mg daily over two months. The patient was able to regain his baseline muscle strength with prednisone treatment alone in three months. Prednisone was then tapered over one month. 

## Discussion

Proximal myopathy presents as symmetrical weakness of proximal upper and/or lower limbs. The underlying etiology is very broad and should consider drugs, alcohol, thyroid disease, osteomalacia, idiopathic inflammatory myopathies (IIM), hereditary myopathies, malignancy, infections, and sarcoidosis [3]. Necrotizing autoimmune myopathy (NAM) accounts for 19% of inflammatory myopathies [1].

Myalgia can occur in up to 10% of patients prescribed statin but myopathies are still rare. The increasing use of statin in higher doses will likely increase the occurrence of these rare side effects and further preventive measures and studies are warranted [4].

Unlike the self-limiting statin myopathy, SINAM is much more severe and is associated with significant symmetric proximal muscle weakness, markedly elevated CK level, and persistent symptoms despite statin discontinuation. The pathophysiology of SINAM is still a topic of discussion but it is thought to be secondary to statin-induced autoimmunity against HMG-CoA, which is the rate-limiting enzyme involved in cholesterol synthesis [5]. Studies have suggested genetic susceptibility with host immunogenicity such as specific class II human leukocyte antigen (HLA) alleles, i.e.HLA-DRB1*11: 01 and 07: 01, along with environmental triggers like a statin or mushrooms [6].

Diagnosis of SINAM can be tricky as the onset is highly variable [7]. It is important to distinguish statin-induced myopathy from self-limited myopathies as the treatment may include immunosuppressive modalities [8]. The first step would be proper history and examination. Medications that could have interaction with a statin to increase its toxicity should be scrutinized. There should be a good baseline motor power assessment to monitor the progression and response of the treatment. Other causes of proximal myopathy as mentioned earlier should also be explored.

Anti-HMGCR antibodies are present in almost all cases of SINAM [1]. A biopsy would assist to make a proper diagnosis of myopathy featuring necrotizing myopathy or inflammatory infiltrate. The predominant finding was a necrotizing myopathy with minor infiltrates which were confined to perivascular sites in perimysium. The cellular infiltrates mostly demonstrated macrophages [9].

Treatment for SINAM has been a challenge as discontinuation of statin would neither limit the ongoing process of inflammation nor improve the clinical symptoms. The lack of improvement following discontinuation of statins, the need for immunosuppressive therapy, and frequent relapse when treatment was tapered suggest an immune‐mediated etiology for this rare, statin‐associated necrotizing myopathy [10]. Rechallenge with a statin is unsuccessful in most cases [11]. Immunosuppressive treatment is essential and studies regarding intravenous immune globulin (IVIG) treatment are also under investigation. Steroid use is the first line of management and other immunosuppressants like methotrexate, mycophenolate, azathioprine, and rituximab have been used based on severity and physician’s preference [12]. There should be more trials regarding treatment strategy and head-to-head comparison of various treatments for the management of SINAM.

## Conclusions

Statin-induced necrotizing autoimmune myopathy should be suspected in patients who present with proximal myopathy and have markedly elevated creatinine kinase which does not get better with discontinuation of statin. It is important to differentiate it from self-limited statin-induced myopathy as it may require aggressive immunosuppressive treatment.

## References

[1] Sharma P, Timilsina B, Adhikari J, Parajuli P, Dhital R, Tachamo N (2019). Statin-induced necrotizing autoimmune myopathy: an extremely rare adverse effect from statin use. J Community Hosp Intern Med Perspect.

[2] Mammen AL, Pak K, Williams EK, Brisson D, Coresh J, Selvin E, Gaudet D (2012). Rarity of anti-3-hydroxy-3-methylglutaryl-coenzyme A reductase antibodies in statin users, including those with self-limited musculoskeletal side effects. Arthritis Care Res (Hoboken).

[3] Suresh E, Wimalaratna S (2013). Proximal myopathy: diagnostic approach and initial management. Postgrad Med J.

[4] Joy TR, Hegele RA (2009). Narrative review: statin-related myopathy. Ann Intern Med.

[5] Mammen AL (2016). Statin-associated autoimmune myopathy. N Engl J Med.

[6] Mohassel P, Mammen AL (2021). Anti-HMGCR myopathy. J Neuromuscul Dis.

[7] Klein M, Mann H, Pleštilová L, Zámečník J, Betteridge Z, McHugh N, Vencovský J (2015). Increasing incidence of immune-mediated necrotizing myopathy: single-centre experience. Rheumatology (Oxford).

[8] Kanth R, Shah MS, Flores RM (2013). Statin-associated polymyositis following omeprazole treatment. Clin Med Res.

[9] Kassardjian CD, Lennon VA, Alfugham NB, Mahler M, Milone M (2015). Clinical features and treatment outcomes of necrotizing autoimmune myopathy. JAMA Neurol.

[10] Grable-Esposito P, Katzberg HD, Greenberg SA, Srinivasan J, Katz J, Amato AA (2010). Immune-mediated necrotizing myopathy associated with statins. Muscle Nerve.

[11] Nazir S, Lohani S, Tachamo N, Poudel D, Donato A (2017). Statin-associated autoimmune myopathy: a systematic review of 100 cases. J Clin Rheumatol.

[12] Tiniakou E (2020). Statin-associated autoimmune myopathy: current perspectives. Ther Clin Risk Manag.

